# Real-world effectiveness of central dialysis fluid delivery system (CDDS) in maintenance hemodialysis: a retrospective multicenter study

**DOI:** 10.1080/0886022X.2026.2677280

**Published:** 2026-08-04

**Authors:** Yashuang Luo, Wendi Cheng, Ruimin Li, Yuyan Fu, Ying Li, Jun Xue, Yin Zheng, Haiyin Wang

**Affiliations:** a Department of Health Technology Assessment, Shanghai Health Development Research Center (Shanghai Medical Information Center), Shanghai, P.R. China; b Department of Statistics and Data Science, Southern University of Science and Technology, ShenZhen, P.R. China; c Department of Nephrology, Jiading District Central Hospital Affiliated Shanghai University of Medicine & Health Sciences, Shanghai, P.R. China; d Department of Nephrology, Huashan Hospital Affiliated to Fudan University, Shanghai, P.R. China

**Keywords:** Hemodialysis, central dialysis delivery system, nutritional status, inflammation, real-world evidence

## Abstract

This study aimed to compare the real-world effectiveness of a central dialysis fluid delivery system (CDDS) with single-patient dialysis delivery systems (SPDDS) in patients undergoing maintenance hemodialysis (MHD), focusing on inflammation markers, nutritional status, and dialysis adequacy. We conducted a retrospective cohort analysis of 368 MHD patients from three hemodialysis centers in Shanghai, China, between January 2020 and June 2023 was analyzed (CDDS, *n* = 253; SPDDS, *n* = 115). Generalized linear mixed-effects models were used to evaluate longitudinal associations between dialysate delivery systems and clinical outcomes. Subgroup analyses were performed to examine effect modification by age, diabetes status, baseline inflammation, and dialysis modality. Compared with SPDDS, CDDS was associated with higher levels of total protein (β = 0.91 g/L), albumin (β = 1.71 g/L), and hemoglobin (β = 3.50 g/L) after multivariable adjustment (all *p* < 0.05), while no statistically significant differences were observed in creatinine or urea reduction after adjustment. Subgroup analysis indicated that CDDS was particularly effective in improving albumin levels among patients aged < 70 years, those with diabetes, or those with elevated CRP levels. In patients aged ≥ 70, CDDS was associated with better anemia control and enhanced dialysis adequacy. Nutritional benefits were consistently observed in patients receiving a regimen of twice-weekly high-flux hemodialysis combined with once-weekly hemodiafiltration. In conclusion, CDDS use was associated with improved nutritional and hematologic indicators in this real-world cohort, while differences in inflammatory markers and dialysis adequacy were not statistically significant after multivariable adjustment.

## Introduction

The worldwide prevalence of maintenance hemodialysis (MHD) continues to rise, reaching approximately 3.9 million individuals in 2023, with an annual growth rate of 5-7% in low-and middle-income countries [1,2]. Although kidney transplantation is recognized as the most effective treatment for end-stage renal disease (ESRD), its clinical application limited by donor shortages and prolonged waiting times. Consequently, HD remains the predominant life-sustaining therapy for most patients.

Dialysis adequacy depends on three key technical factors, such as a high-performance dialyzer, consistent blood flow and ultrapure dialysis fluid delivered at a precisely controlled rate. Among these, the dialysate and its delivery system play a central, yet often underrecognized, role in ensuring dialysis adequacy and patient safety. Suboptimal microbiological purity contributes to micro-inflammation, electrolyte disturbances, and increased risk of hospitalization and mortality [3,4]. Therefore, standardized production and delivery of high-quality dialysate are essential.

Currently, two main dialysate delivery systems are employed in clinical practice, the single-patient dialysis delivery system (SPDDS), which prepares individualized dialysate in real time at the bedside, and the central dialysis fluid delivery system (CDDS), in which dialysate is produced centrally and distributed simultaneously to multiple treatment station *via* a closed-loop circuit. While SPDDS offers flexibility in use, it may lead to inconsistent dialysate quality due to variations in equipment performance. In contrast, CDDS allows for centralized preparation, real-time quality monitoring, and standardized disinfection. These features confer CDDS advantages in operational efficiency, cost-effectiveness, and infection control, contributing to its growing adoption in high-volume dialysis centers across China [5,6]. In regions with large dialysis populations and high clinical workloads, these advantages make CDDS especially valuable as a core component in ensuring safe and effective hemodialysis.

Several studies have compared CDDS and SPDDS in terms of dialysate purity and their impact on inflammation. Kara et al. [7] reported that CDDS implementation significantly reduced endotoxin levels and improved infection control in HD centers. Similarly, Tawfik et al. [8] found that CDDS equipped with multi-stage endotoxin filters and daily ozone disinfection could maintain endotoxin levels below 0.001 EU/mL and reduce C-reactive protein (CRP) by 37%. These studies suggest that CDDS may attenuate inflammation, as lower dialysate endotoxin levels are associated with decreased serum interleukin-6 and a slower rise in β2-microglobulin [7]. Recent real-world studies have further highlighted the prognostic significance of malnutrition inflammation complex syndrome (MICS) and its link to adverse clinical outcomes in maintenance HD patients [8,9].

However, few studies have systematically evaluated whether CDDS influences broader clinical outcomes or whether specific patient subgroups, such as older adults or individuals with diabetes, derive differential benefit from its use. To address this gap, the current study aimed to comprehensively evaluate the impact of CDDS versus SPDDS on systemic inflammation, nutritional status, anemia control, and dialysis adequacy in a retrospective cohort of maintenance HD patients, using real-world data from three dialysis centers in Shanghai, China. Additionally, we performed subgroup analyses to identify patients who may benefit from CDDS, providing new insights into personalized dialysis practice.

## Methods

### Study design and data source

This retrospective, multicenter, real-world cohort study was conducted to compare the long-term effectiveness and safety of the CDDS versus the SPDDS in patients with ESRD. Data were obtained from three large hemodialysis centers in Shanghai, where both systems are routinely used in clinical practice, thus providing an appropriate setting for comparative evaluation. The study period was from January 1, 2020, to June 30, 2023.

The study protocol was reviewed and approved by the Ethics Committee of the Shanghai Health Development Research Center (Approval No. 2023002) and was conducted in accordance with the Declaration of Helsinki. As a retrospective observational study using de-identified clinical data, informed consent for the use of medical records in research had been obtained from all participants at the time of enrollment. Data entry was performed independently by two trained researchers using a double-entry protocol. Discrepancies between the datasets were resolved by a third investigator by referring to the original medical records. Outliers were defined as values falling outside clinically acceptable ranges (e.g., hemoglobin < 50 or > 180 g/L) or exceeding three standard deviations from the cohort mean. Inconsistencies referred to logically incompatible entries, such as post-dialysis creatinine levels exceeding pre-dialysis values. Logical consistency checks were conducted to validate the sequence of events (e.g., dialysis initiation dates preceding follow-up) and internal coherence across related clinical variables (e.g., hemoglobin trends and erythropoietin doses). These procedures ensured data integrity prior to analysis.

### Study population

Patients with ESRD receiving maintenance hemodialysis at the three participating centers were considered for inclusion. Eligible participants had to meet the following criteria: (1) confirmed diagnosis of stage 5 chronic kidney disease (CKD) and undergoing maintenance HD; (2) receipt of regular HD for at least three months prior to the study start, following a standard regimen of 3 sessions per week, each lasting approximately four hours; (3) use of dialyzers with membrane surface areas approximately ranging from 1.5 to 1.8 m^2^, consistent with devices commonly used in routine clinical practice [10]; and (4) treatment with one of the following dialysis modalities: twice-weekly high-flux HD (HFHD) combined with once-weekly hemodiafiltration (HDF), thrice-weekly HFHD, thrice-weekly intermittent infusion HDF (iHDF), or thrice-weekly low-flux HD (LFHD). Exclusion criteria were (1) diagnosis of acute kidney injury or use of temporary dialysis; (2) incomplete medical records or significant loss to follow-up (defined as missing more than 40% of required follow-up data points); (3) kidney transplantation during the study period; (4) any switch in dialysis fluid delivery system (i.e., from CDDS to SPDDS or vice versa); or (5) concurrent use of other renal replacement therapies. Based on these criteria, 368 patients were included in the final analysis.

### Group allocation and study outcomes

Based on the type of dialysate delivery system used in routine clinical care, patients were retrospectively assigned to either the CDDS group (*n* = 253) or the SPDDS group (*n* = 115). Group assignment was nonrandomized and determined by standard clinical practice. To minimize potential selection bias, relevant confounders were adjusted for in subsequent statistical analyses. Additionally, a predefined subgroup of patients receiving a consistent dialysis regimen (specifically, twice-weekly HFHD combined with once-weekly HDF) was analyzed separately to isolate the effect of the dialysate delivery system from that of dialysis modality.

The primary outcomes comprised dialysis adequacy (assessed by single-pool Kt/V), inflammation (C-reactive protein (CRP) levels), hemoglobin levels, nutritional status (evaluated *via* total protein and serum albumin levels). Secondary outcomes included the session-level incidence of intradialytic hypotension, the requirement for erythropoiesis-stimulating agents (ESAs), and use of anticoagulants. SpKt/V calculated according to the second-generation Daugirdas formula [11]. Post-dialysis blood samples were obtained using the slow-flow technique to minimize access recirculation, in which the blood flow rate was reduced to approximately 50–100 mL/min for 15–30 s before sampling from the arterial port of the extracorporeal circuit.

Intradialytic hypotension was defined as a decrease in systolic blood pressure ≥ 20 mmHg during dialysis accompanied by symptoms requiring clinical intervention. The incidence of intradialytic hypotension was analyzed at the session level and was calculated as the proportion of dialysis sessions complicated by at least one hypotensive episode during the follow-up period.

All clinical and laboratory data were obtained from routine monitoring records. Laboratory tests were conducted by certified laboratories at each center under standardized quality management practices in Shanghai across the participating centers [10,12]. Laboratory measurements were summarized at 3-month intervals throughout the follow-up period, resulting in repeated measurements for each patient. Dialysis machines used in the SPDDS group were from established manufacturers, including Fresenius Medical Care AG & Co. KGaA (Germany), Baxter International Inc. (USA), and Shandong Weigao Group Medical Polymer Co., Ltd. (China), while machines in the CDDS group were supplied by JMS Co., Ltd. (Japan). All machines underwent maintenance and disinfection in accordance with manufacturer and facility protocols.

### Statistical analysis

Intergroup comparisons of baseline characteristics were performed with descriptive statistics. Categorical variables were expressed as frequencies (percentages) and intergroup differences were examined utilizing the chi-squared test. For continuous variables, distribution normality was assessed *via* the Shapiro-Wilk test. Normally distributed data are presented as mean ± standard deviation (SD) and were compared using independent t-tests; non-normally distributed data are presented as median (interquartile range, IQR) and were compared using the Mann-Whitney U test. Baseline characteristics of the modality-restricted subgroup were summarized using the same methods as in the primary analysis to assess comparability between groups.

Associations between dialysate delivery systems and clinical outcomes were evaluated using generalized linear mixed-effects models. Covariates were selected *a priori* based on clinical relevance and previous literature (e.g., age, sex, and blood flow rate), which is recommended in epidemiological and clinical modeling to improve model stability and reduce biased inference [13]. Time was modeled as a continuous variable representing 3-month intervals, and a time × group interaction term was included to assess whether temporal trends differed between CDDS and SPDDS. Patient-level random intercepts were included to account for within-patient correlation arising from repeated measurements over time.

Exploratory subgroup analyses were performed according to age, diabetes status, baseline CRP level, and dialysis modality to explore potential heterogeneity in associations. Within each subgroup, analyses were conducted using the same modeling approach as in the primary analysis. These analyses were not adjusted for multiple comparisons and should therefore be interpreted as hypothesis-generating.

In addition, because dialysis modality was moderately associated with the dialysate delivery system (Cramér’s *V* = 0.35), it was not included as a covariate in the primary multivariable models to avoid potential multicollinearity. Instead, a predefined modality-restricted sensitivity analysis was conducted among patients receiving the same dialysis regimen, namely HFHD combined with HDF, to evaluate the robustness of the primary findings under comparable modality conditions.

Missing data were handled using multiple imputation by chained equations (MICE) under the missing-at-random assumption. The proportion of missing values was generally low (< 10% across variables) and was mainly due to occasional missed laboratory measurements during routine follow-up. The imputation model included all variables used in the primary analyses, including dialysate delivery system, age, sex, dialysis vintage, blood flow rate, dialyzer membrane surface area, outcome variables, and the time variable representing 3-month intervals. Five imputed datasets were generated and pooled using Rubin’s rules [14,15]. All statistical analyses were conducted in R (version 4.2.0), with a two-tailed *p*-value < 0.05 considered statistically significant.

## Results

### Baseline characteristics

A total of 368 on MHD patients were included in the final analysis (CDDS: *n* = 253; SPDDS: *n* = 115). The median dialysis vintage was 3 years. As summarized in Table 1, the two groups were comparable in terms of key baseline characteristics. The mean age was 62 years in the CDDS group and 64 years in the SPDDS group, with a comparable proportion of male patients (64.4% vs. 66.1%). No significant intergroup differences were observed in body weight, blood flow rate, dialyzer membrane surface area (all *p* > 0.05), or in the distribution of primary kidney diseases, including diabetic nephropathy, glomerulonephritis, and hypertensive nephropathy (*p* = 0.181). Baseline laboratory parameters, such as serum calcium, phosphate, potassium, and intact parathyroid hormone (iPTH) levels, were also similar between groups. The baseline CRP levels did not differ significantly between the CDDS and SPDDS groups (6.69 ± 1.41 mg/L vs. 5.80 ± 1.21 mg/L; *p* = 0.214), as shown in Table 1.

**Table 1.** Baseline characteristics of patients receiving CDDS or SPDDS.

Characteristic	CDDS (*n* = 253)	SPDDS (*n* = 115)	*P* value
Age, years	62 ±12.42	64 ± 13.04	0.098
Weight, kg	61.75 ±12.19	62.76 ±11.85	0.461
Blood flow rate, mL/min	235.93 ± 16.11	236.38 ±16.46	0.805
Dialyzer membrane area, m^2^	1.64 ± 0.12	1.66 ± 0.11	0.761
Total protein, g/L	63 (59–68)	64 (60–69)	0.536
Albumin, g/L	36 (33–40)	36 (34–40)	0.780
Hemoglobin, g/L	82 (70–96)	85 (72–98)	0.256
C-reactive protein, mg/L	6.69 ± 1.41	5.80 ± 1.21	0.214
Serum calcium, mmol/L	2.14 ± 0.57	2.01 ± 0.64	0.107
Serum phosphorus, mmol/L	1.66 ± 0.79	1.28 ± 0.91	0.139
Serum potassium, mmol/L	3.22 ± 1.30	3.84 ± 2.51	0.090
iPTH, pg/mL	280.96 ± 58.51	243.23 ± 60.90	0.271
Dialysis vintage, years	3 (2–6)	3 (2–4)	0.169
Sex			0.848
Male	163 (64.40)	76 (66.12)	
Dialysis modality			<0.001***
2 HFHD + 1 HDF per week	53 (20.94)	54 (47.01)	
3 HFHD per week	172 (68.06)	9 (7.77)	
3 IHDF per week	28 (11.10)	0 (0.00)	
3 LFHD per week	0 (0.00)	52 (45.22)	
Primary disease			0.181
Diabetic nephropathy	64 (25.30)	33 (28.70)	
Primary glomerular disease	37 (14.62)	25 (21.74)	
Hypertensive nephropathy	42 (16.60)	17 (14.78)	
Other secondary glomerular diseases	12 (4.74)	8 (6.96)	
Others	98 (38.74)	33 (27.83)	

Abbreviations: CDDS, central dialysis fluid delivery system; SPDDS, single-patient dialysis fluid delivery system; HDF, hemodiafiltration; HFHD, high-flux hemodialysis; IHDF, intermittent infusion hemodiafiltration; LFHD, low-flux hemodialysis; PTH, parathyroid hormone; Note: *** indicate *p* < 0.001.

Dialysis modality differed significantly between groups (*p* < 0.001). While the majority (68%) of patients in the CDDS group were on a thrice-weekly HFHD schedule, the SPDDS group showed a more varied distribution, with LFHD (45%) and a twice-weekly HFHD plus once-weekly HDF regimen (47%) being the predominant modalities. The association between the dialysis delivery system and dialysis modality was moderate, as indicated by Cramér’s *V* = 0.35.

### Nutritional and hemoglobin

Compared to the SPDDS group, the CDDS group exhibited significantly higher mean levels of total protein, serum albumin, and hemoglobin (all *p* < 0.05; Table 2). In multivariable linear regression models adjusting for age, sex, and dialysis vintage, the use of CDDS remained independently associated with these favorable outcomes (Table 3). The use of CDDS was associated with a significant increase in total protein (β = 0.91 g/L, *p* < 0.05), serum albumin (β = 1.71 g/L, *p* < 0.001), and hemoglobin levels (β = 3.50 g/L, *p* < 0.001).

**Table 2.** Univariate comparison of clinical outcomes between CDDS and SPDDS group.

Indicator	CDDS (*N* = 253)	SPDDS (*N* = 115)	*P* value
	Mean ± SE	Mean ± SE	
Nutrition			
Total Protein, g/L	68.69 ± 4.73	67.63 ± 5.13	0.001***
Albumin, g/L	41.95 ± 6.82	39.30 ± 5.36	<0.001***
Hemoglobin, g/L	108.93 ± 10.21	104.92 ± 11.57	0.04
Dialysis adequacy			
ΔCreatinine, μmol/L	571.80 ± 180.93	536.22 ± 186.57	<0.001***
ΔUrea, mmol/L	18.25 ± 7.60	16.16 ± 6.56	<0.001***
Kt/V	1.39 ± 0.48	1.27 ± 0.38	0.16
Safety			
CRP, mg/L	6.72 ± 1.16	10.84 ± 2.02	0.017**
Session-level intradialytic hypotension incidence, %	20.6	35.7	0.36
Medication usage			
Anticoagulant, IU/session	3201.02 ± 1628.22	3373.87 ± 1478.77	0.33
Erythropoietin, IU/patient/month	73057.85 ±52928.73	84340.44 ± 53516.97	0.06

Abbreviations: ΔCreatinine and ΔUrea refer to pre- and post-dialysis concentration differences; CRP, C-reactive protein; IU, international unit; CDDS, central dialysis fluid delivery system; SPDDS, single-patient dialysis fluid delivery system.

Note.

* *p* < 0.05.

** *p* < 0.01.

*** *p* < 0.001.

**Table 3.** Multivariable regression analysis of post-dialysis outcomes considering time effects.

Outcome	β (SE)	95% CI	*p* value	Conditional R²	Marginal R²
Total protein (g/L)	0.91 (0.38)	(0.17, 1.65)	0.017*	0.786	0.779
Albumin (g/L)	1.71 (0.43)	(0.87, 2.55)	<0.001***	0.211	0.205
Hemoglobin (g/L)	3.50 (0.95)	(1.64, 5.36)	<0.001***	0.587	0.581
CRP (mg/L)	0.28 (0.21)	(−0.13, 0.69)	0.18	0.507	0.498
ΔCreatinine (μmol/L)	16.77 (18.99)	(−20.45, 53.99)	0.38	0.312	0.288
ΔUrea (mmol/L)	1.17 (0.71)	(−0.22, 2.56)	0.10	0.187	0.159

Abbreviations: CRP, C-reactive protein; ΔCreatinine and ΔUrea refer to pre- and post-dialysis concentration differences; CDDS, central dialysis fluid delivery system; SPDDS, single-patient dialysis fluid delivery system. Notes: *p < 0.05, **p < 0.01, ***p < 0.001. Regression coefficients were estimated using generalized linear mixed-effects models. Patient ID was included as a random effect to account for repeated measurements. Model fit is presented as marginal and conditional *R*^2^. Models were adjusted for age, sex, dialysis vintage, blood flow rate, and dialyzer membrane surface area.

### Inflammation and solute clearance markers

During follow-up, the mean CRP level was lower in the CDDS group than in the SPDDS group in the unadjusted analysis (6.72 ± 1.16 mg/L vs. 10.84 ± 2.02 mg/L; *p* = 0.017). However, these differences were attenuated and were no longer statistically significant after multivariable adjustment. The CDDS group also demonstrated significantly greater post-dialysis reductions in serum creatinine and blood urea nitrogen (both *p* < 0.001). No difference was found for dialysis adequacy measured by spKt/V (*p* > 0.05). After adjustment for age, sex, dialysis vintage, body weight, blood flow rate, dialyzer surface area, follow-up time, and the time-group interaction, the differences in CRP, creatinine, and urea levels between the two groups were no longer statistically significant (all *p* > 0.05; Table 3).

### Intradialytic hypotension and medication usage

No significant differences were observed between the groups in safety outcomes or medication usage (Table 2). The session-level incidence of intradialytic hypotension was 20.6% in the CDDS group and 35.7% in the SPDDS group (*p* = 0.36). Both groups received comparable mean doses of low-molecular-weight heparin per session (CDDS: 3201 IU; SPDDS: 3374 IU) and erythropoiesis-stimulating agent (ESA) per patient per month (CDDS: 73,057 IU; SPDDS: 84,340 IU).

### Subgroup analyses

Subgroup analyses were undertaken to evaluate whether the effects of CDDS differed across specific patient populations (Table 4).

**Table 4.** Subgroup analyses of the association between dialysis delivery system and post-dialysis clinical outcomes.

Subgroup	Total protein (g/L)	Albumin (g/L)	Hemoglobin (g/L)	CRP (mg/L)	ΔCreatinine (μmol/L)	ΔUrea (mmol/L)
	β (SE)	β (SE)	β (SE)	β (SE)	β (SE)	β (SE)
Dialysis modality						
2 HFHD + 1 HDF per week	**0.96 (0.41)** *	**7.11 (0.63)** ***	**8.46 (1.30)** ***	−2.34 (1.34)	−17.68 (19.8)	**−3.32 (1.13)** **
Age group						
<70	**0.89 (0.58)** *	**0.93 (0.18)** ***	**4.70 (0.59)** ***	−0.94 (0.40)	**20.18 (10.87)** *	0.15 (0.33)
≥70	0.45 (0.23)	**0.71 (0.29)** *	**3.54 (0.80)** ***	−1.42 (0.83)	**49.24 (10.19)** ***	0.69 (0.48)
With or without diabetes						
With diabetes	**0.87 (0.26)** ***	**0.58 (0.21)** **	**3.20 (0.85)** ***	−0.37 (0.22)	**33.97 (11.46)** **	**0.78 (0.31)** *
Without diabetes	**0.28 (0.13)** *	**0.98 (0.16)** ***	**2.25 (0.44)** ***	−0.63 (0.34)	**12.42 (6.15)** *	0.10 (0.24)
Baseline CRP level						
High CRP (>5mg/L)	0.40 (0.48)	**1.79 (0.63)** **	**4.66 (1.55)** **	−3.02 (2.46)	**45.06 (18.32)** *	**1.49 (0.72)** *
Low CRP (≤5mg/L)	**0.41 (0.12)** ***	**0.91 (0.13)** ***	**1.99 (0.40)** ***	−0.05 (0.05)	**13.96 (5.62)** *	0.05 (0.21)

Abbreviations: CRP, C-reactive protein; ΔCreatinine and ΔUrea refer to pre- and post-dialysis concentration differences; HFHD, high-flux hemodialysis; HDF, hemodiafiltration; Notes.

* *p* < 0.05.

** *p* < 0.01.

*** *p* < 0.001. Values are expressed as β coefficients (standard error) from subgroup-specific multivariable regression models comparing CDDS versus SPDDS.

To mitigate the confounding effect of differing dialysis modalities, we evaluated outcomes exclusively within the cohort of patients receiving the identical regimen of twice-weekly HFHD plus once-weekly HDF. Baseline characteristics of the modality-restricted subgroup are presented in Supplementary Table S1. The two groups were generally comparable across measured covariates. Within this modality-matched subgroup, the use of CDDS remained significantly associated with higher serum albumin serum albumin (β = 7.11, *p* < 0.001), hemoglobin level (β = 8.46, *p* < 0.001), and greater urea reduction (β = −3.32, *p* = 0.01) compared to SPDDS. In additional analyses adjusting for dialysis modality, the effect estimates for CDDS were attenuated across several outcomes, although the overall direction of association for albumin and hemoglobin remained consistent with the primary analysis (Supplementary Table S2). Crucially, however, within this restricted cohort, the previously observed differences in C-reactive protein (CRP) levels and dialysis adequacy (spKt/V) were completely attenuated (all *p* > 0.05).

When stratified by age, patients under 70 years old experienced improvements in albumin (β = 0.93, *p* < 0.001), CRP (β = −1.04, *p* < 0.001), and hemoglobin (β = 4.70, *p* < 0.001). Among patients aged 70 years or older, CDDS use was associated with increased hemoglobin (β = 3.54, *p* < 0.001) and better dialysis adequacy (ΔCreatinine, ΔUrea), whereas CRP levels showed no significant change.

In subgroup analyses of patients with comorbidities, those with diabetes mellitus exhibited significant improvements following CDDS treatment: total protein increased (β = 0.87, *p* < 0.001), hemoglobin levels rose (β = 3.20, *p* < 0.001), and serum creatinine decreased (β = −33.97, *p* = 0.002). Conversely, in the subgroup with elevated systemic inflammation at baseline (defined as high CRP levels), CDDS utilization was linked to significant increases in serum albumin (β = 1.79, *p* < 0.05), hemoglobin (β = 4.66, *p* < 0.05), and greater reductions in creatinine (β = 45.06, *p* = 0.02).

## Discussion

This multicenter, real-world retrospective cohort study comprehensively evaluated the clinical effectiveness of CDDS compared to SPDDS in patients on MHD. Our findings demonstrate that CDDS is independently associated with improved nutritional status and hematologic parameters, notably serum albumin and hemoglobin levels, while maintaining equivalent dialysis adequacy and a comparable safety profile. These results extend the scope of earlier single-center studies that primarily reported short-term biochemical benefits, thus underscoring the broader clinical value of centralized dialysate delivery systems in routine hemodialysis care.

In unadjusted analyses, patients receiving CDDS showed lower average CRP levels and greater urea/creatinine clearance. In the adjusted analyses, the differences in CRP and solute reduction parameters between groups were attenuated and did not reach statistical significance. Therefore, the findings should not be interpreted as evidence that CDDS independently reduces inflammatory status or improves dialysis adequacy. Although the intergroup difference in Kt/V was not statistically significant, the findings highlight the complexity of interpreting different indicators of solute removal in clinical practice. Absolute reductions in solute concentrations (ΔUrea and ΔCreatinine) may differ between groups even when the normalized adequacy index spKt/V remains comparable. This difference reflects the distinct measurement properties of absolute concentration changes versus normalized clearance indices, as spKt/V represents urea clearance normalized to the patient’s distribution volume and incorporates treatment time and ultrafiltration parameters. Previous studies have emphasized that dialysis quality and patient outcomes are influenced by multiple technical and biological factors, including dialysate purity, treatment stability, and patient nutritional status, which may not be fully captured by a single adequacy metric such as spKt/V [16,17].

Even after adjustment for key confounders (including age, sex, dialysis vintage, body weight, blood flow rate, and dialyzer surface area), CDDS remained independently associated with higher serum albumin and hemoglobin levels, suggesting a sustained benefit in supporting protein-energy balance and anemia management. In the modality-matched analysis, differences in CRP and dialysis adequacy were attenuated and did not reach statistical significance. This finding suggests that the observed associations between CDDS and improved albumin and hemoglobin levels are unlikely to be explained solely by differences in systemic inflammation or small-solute clearance. Serum albumin in maintenance hemodialysis patients reflects multiple processes, including nutritional intake, comorbidity burden, fluid status, and inflammatory activity, and should not be interpreted as a single process or as a direct indicator of nutritional improvement alone [18]. Hemoglobin levels are likewise influenced by multiple determinants, including nutritional status, inflammation, and anemia-related treatment responsiveness [19]. In addition, CRP may not fully capture subtle or chronic inflammatory states in hemodialysis patients [20]. Therefore, these findings should be interpreted as observational associations rather than evidence of a specific mechanistic effect, and the underlying biological mechanisms require further investigation. Inclusion of dialysis modality in the multivariable models resulted in attenuation of effect estimates, supporting its role as an important confounder; however, the persistence of directionally consistent associations suggests that modality differences alone may not fully explain the observed findings.

The clinical benefits of CDDS were most evident in specific subgroups, including patients younger than 70 years and those with diabetes or elevated baseline CRP, who showed greater improvements in albumin and hemoglobin. These findings may reflect greater metabolic responsiveness in younger patients [21], as well as the amplified impact of high-purity dialysate in individuals with inflammatory or malnutrition. Among older patients (≥ 70 years), although inflammatory markers did not change significantly, CDDS was associated with improved dialysis adequacy and hemoglobin levels. This may be attributed to the greater consistency in dialysate composition and delivery stability provided by centralized systems [22,23].

One possible explanation for these observations may relate to differences in dialysate purity and fluid distribution systems reported in previous studies of centralized dialysis delivery systems; however, dialysate endotoxin levels were not directly measured in the present study, and therefore mechanistic interpretations remain hypothetical [24,25]. Impurities in dialysate can active monocytes and stimulate IL-6-mediated hepatic production of CRP, contributing to the inflammation malnutrition anemia (IMA) cycle in MHD patients [26]. CDDS, by standardizing dialysate purity and minimizing contamination risk, therefore help interrupt this pathogenic cascade, particularly in vulnerable subgroups [27].

In addition to its clinical impact, CDDS may offer operational and economic advantages. Centralized fluid production supports batch preparation, automated disinfection, and real-time quality monitoring, which could reduce labor demands, enhanced safety, and lower costs in large-scale dialysis centers [5]. Although our study did not directly assess economic outcomes, the observed increase in hemoglobin without a corresponding rise in erythropoiesis-stimulating agent (ESA) use suggests a potential for cost-savings, which can be supported by prior cost-modeling research.

Several limitations of this study should be acknowledged. First, its retrospective design might introduce residual confounding, as unmeasured factors (e.g., dietary protein intake, inflammatory control strategies, socioeconomic status) were not adjusted for. Although the adjusted associations observed for albumin and hemoglobin were modest in magnitude, it should be interpreted cautiously in the context of this observational study. Second, the nonrandomized assignment of dialysis modality could bias results, and although we conducted regimen-matched subgroup analyses, unobserved center-level differences (e.g., infrastructure, staff expertise) could influence outcomes. Because dialysate delivery systems were used according to routine practice patterns at participating centers, center-level practice differences and equipment characteristics may have contributed to residual confounding. Residual confounding related to patient case-mix and nonrandom dialysis modality allocation cannot be fully excluded despite multivariable adjustment and modality-restricted analyses. In addition, some subgroup effect estimates were relatively large, which may reflect smaller subgroup sample sizes, limited precision, residual confounding, or model instability. The subgroup analyses were conducted using stratified models to explore potential heterogeneity in associations, were not formally prespecified for confirmatory testing, and were not adjusted for multiple comparisons. Therefore, these subgroup findings should be regarded as exploratory and hypothesis-generating rather than definitive evidence of subgroup-specific effects. Third, we could not evaluate cardiovascular outcomes, hospitalization rates, or patient-reported quality-of-life, which constrain a holistic evaluation of CDDS benefits. Lastly, because the participating centers were all located in Shanghai, a highly urbanized setting with relatively standardized dialysis infrastructure, the external validity of our conclusions for rural or low-resource settings remains uncertain. Despite these limitations, these findings provide real-world evidence regarding the association between dialysate delivery systems and selected clinical indicators in maintenance hemodialysis patients. Further prospective studies with direct measurements of dialysate quality and more rigorous study designs are needed to confirm these observations. The use of longitudinal, multicenter real-world data provides additional context for understanding potential differences associated with dialysate delivery systems in routine clinical practice. These findings may inform future research and quality improvement initiatives in dialysis care.

## Conclusion

This retrospective multicenter cohort study showed that, compared with SPDDS, CDDS use was associated with improved nutritional and hematologic indicators in maintenance hemodialysis patients. However, differences in inflammatory markers and dialysis adequacy were not statistically significant after multivariable adjustment.

## Supplementary Material

Appendix0416 (1).docx

## Data Availability

The data that support the findings of this study are not publicly available due to privacy and institutional data protection regulations. The statistical analysis plan, and the analytic code used for the present study are available from the corresponding author upon reasonable request and with appropriate institutional approval.
