# Supplementary material for: Real-world effectiveness of central dialysis fluid delivery system (CDDS) in maintenance hemodialysis: a retrospective multicenter study
Source: Ren Fail. 2026 Aug 4;48(1):2677280. doi: 10.1080/0886022X.2026.2677280 (PMC13439772; doi:10.1080/0886022X.2026.2677280)
Supplement: Supplementary file 1 [file IRNF_A_2677280_SM6509.docx]

**Appendix 1：**

Supplementary Table 1. Baseline characteristics of patients receiving the same dialysis modality (2HFHD + 1HDF per week), stratified by dialysate delivery system

| **Characteristic** | **CDDS**  **(n=253)** | **SPDDS**  **(n=115）** | **P value** |
| --- | --- | --- | --- |
| **Age, years** | 61±11.8 | 62±12.1 | 0.62 |
| **Weight, kg** | 62.1±11.5 | 61.8±12.0 | 0.88 |
| **Blood flow rate, ml/min** | 236±15 | 234±16 | 0.47 |
| **Dialyzer membrane area, m^2^** | 1.64±0.12 | 1.65±0.11 | 0.71 |
| **Total Protein, g/L** | 63 (59–68) | 64 (60–69) | 0.53 |
| **Albumin, g/L** | 36 (33–40) | 36 (34–40) | 0.76 |
| **Hemoglobin, g/L** | 83 (70–95) | 84 (72–97) | 0.69 |
| **C-Reactive Protein, mg/L** | 6.5±13.8 | 6.1±12.5 | 0.82 |
| **Serum calcium, mmol/L** | 2.12±0.56 | 2.05±0.60 | 0.48 |
| **Serum phosphorus, mmol/L** | 1.62±0.78 | 1.35±0.85 | 0.21 |
| **Serum potassium, mmol/L** | 3.30±1.25 | 3.75±2.40 | 0.29 |
| **iPTH, pg/mL** | 278±60 | 250±58 | 0.18 |
| **Dialysis vintage, years** | 3 (2–6) | 3 (2–5) | 0.57 |
| **Gender** |  |  | 0.79 |
| Male | 34 (64.15) | 33 (61.11) |  |
| **Primary Disease** |  |  | 0.64 |
| Diabetic Nephropathy | 14 (26.42) | 16 (29.63) |  |
| Primary Glomerular Disease | 8 (15.09) | 10 (18.52) |  |
| Hypertensive Nephropathy | 9 (16.98) | 8 (14.81) |  |
| Other Secondary Glomerular Diseases | 3 (5.66) | 4 (7.41) |  |
| Others | 19 (35.85) | 16 (29.63) |  |

Abbreviations: CDDS, central dialysis fluid delivery system; SPDDS, single-patient dialysis fluid delivery system; HDF, hemodiafiltration; HFHD, high-flux hemodialysis; IHDF, intermittent infusion hemodiafiltration; LFHD, low-flux hemodialysis; PTH, parathyroid hormone;

Supplementary Table 2. Fully adjusted generalized linear mixed-effects models evaluating the association between dialysate delivery system and longitudinal outcomes, including dialysis modality as a covariate

| Outcome | β (CDDS vs SPDDS)(SE) | Δβ(%)after adding modality | β (2HFHD+1HDF) (SE) | β (3HFHD) (SE) | β (3IHDF) (SE) | Conditional R² | Marginal R² |
| --- | --- | --- | --- | --- | --- | --- | --- |
| Total protein (g/L) | 0.64 (0.35)** | −29.7% | 0.82 (0.27)** | 0.65 (0.24)** | 0.91 (0.31)** | 0.79 | 0.31 |
| Albumin (g/L) | 1.32 (0.40)** | −22.8% | 1.12 (0.29)*** | 0.88 (0.26)** | 1.35 (0.33)*** | 0.62 | 0.18 |
| Hemoglobin (g/L) | 2.78 (0.90)* | −20.6% | 2.45 (0.80)** | 1.92 (0.75)* | 2.88 (0.90)** | 0.58 | 0.16 |
| CRP (mg/L) | 0.12 (0.20) | −57.1% | −0.95 (0.52) | −0.72 (0.48) | −1.10 (0.60)* | 0.55 | 0.12 |
| ΔCreatinine (μmol/L) | 8.10 (18.10) | −51.7% | 12.40 (10.2) | 9.30 (9.8) | 15.20 (11.5) | 0.52 | 0.10 |
| ΔUrea (mmol/L) | 0.62 (0.68)* | −47.0% | 0.88 (0.55) | 0.70 (0.52) | 1.05 (0.63) | 0.53 | 0.11 |

Abbreviations: ΔCreatinine and ΔUrea refer to pre- and post-dialysis concentration differences; CRP, C-reactive protein; IU, international unit; CDDS, central dialysis fluid delivery system; SPDDS, single-patient dialysis fluid delivery system.

Note: All models were fitted using generalized linear mixed-effects models with patient-level random intercepts and were adjusted for prespecified covariates (age, sex, dialysis vintage, blood flow rate, and dialyzer membrane surface area). Dialysis modality was included as a categorical variable with 3LFHD as the reference category. β coefficients for dialysis modality represent differences compared with the reference group.Δβ (%) indicates the percentage change in the CDDS coefficient after inclusion of dialysis modality, relative to the primary model without modality: Δβ (%) = (β_with modality − β_without modality) / β_without modality×100%. *p < 0.05; **p < 0.01; ***p < 0.001.

**Appendix 2：**

**Statistical Analysis Plan for** **Real-World Effectiveness of Central Dialysis Fluid Delivery System (CDDS) in Maintenance Hemodialysis: A Retrospective Multicenter Study**

**1. Study design and objective**

This study was a retrospective, multicenter observational cohort study using routinely collected clinical data from three hemodialysis centers in Shanghai, China, between January 1, 2020 and June 30, 2023. The primary objective was to evaluate the association between dialysate delivery system and longitudinal clinical outcomes in patients receiving maintenance hemodialysis.

**2. Study population**

Eligible patients were adults with stage 5 chronic kidney disease receiving maintenance hemodialysis for at least 3 months before study entry. Patients were excluded if they had acute kidney injury, temporary dialysis, kidney transplantation during follow-up, a switch in dialysate delivery system during follow-up, concurrent use of other renal replacement modalities, or substantial missing follow-up data.

The final analytic cohort included 368 patients: CDDS group: 253 patients and SPDDS group: 115 patients

**3. Exposure definition**

The primary exposure was the dialysate delivery system used during routine clinical care:**CDDS**: central dialysis fluid delivery system; **SPDDS**: single-patient dialysis delivery system. Exposure was coded as a binary variable in the adjusted analyses: CDDS = 1; SPDDS = 0

**4. Outcome definitions**

**4.1 Primary longitudinal outcomes**

The primary longitudinal outcomes were:

- total protein (g/L)
- serum albumin (g/L)
- hemoglobin (g/L)
- C-reactive protein (CRP, mg/L)
- pre- to post-dialysis creatinine reduction (ΔCreatinine, μmol/L)
- pre- to post-dialysis urea reduction (ΔUrea, mmol/L)

**4.2 Dialysis adequacy**

Single-pool Kt/V (spKt/V) was examined descriptively in the unadjusted analyses. Because no significant between-group difference was observed in the primary unadjusted comparison, spKt/V was not included in the final multivariable mixed-effects outcome table.

**4.3 Secondary outcomes**

Secondary outcomes included:

- session-level intradialytic hypotension incidence
- anticoagulant dose per session
- erythropoietin use per patient per month

**5. Definition of intradialytic hypotension**

Intradialytic hypotension was defined as a decrease in systolic blood pressure of at least 20 mmHg during dialysis accompanied by symptoms requiring clinical intervention.

This outcome was analyzed at the **session level** and expressed as the proportion of dialysis sessions complicated by at least one hypotensive episode during follow-up.

**6. Time structure**

Clinical and laboratory measurements were summarized at **3-month intervals** throughout follow-up. Time was modeled as a continuous variable representing 3-month intervals from cohort entry.

**7. Covariates**

Covariates were selected **a priori** on the basis of clinical relevance and previous literature, rather than automated variable-selection procedures. The adjusted models included:

- age
- sex
- dialysis vintage
- blood flow rate
- dialyzer membrane surface area

Body weight was additionally included for selected outcomes where clinically appropriate.

Dialysis modality was not included in the primary multivariable models because it was moderately associated with dialysate delivery system (Cramér’s V = 0.35), which raised concern for multicollinearity and unstable effect estimation. Modality-related confounding was instead addressed through a predefined modality-restricted sensitivity analysis.

**8. Baseline analyses (Table 1)**

Baseline characteristics were summarized by exposure group.

Normally distributed continuous variables were presented as mean ± standard deviation and compared using independent t-tests. Non-normally distributed continuous variables were presented as median (interquartile range) and compared using Mann–Whitney U tests. Categorical variables were presented as frequencies (percentages) and compared using chi-squared tests.

The association between dialysate delivery system and dialysis modality was additionally summarized using Cramér’s V.

**9. Unadjusted outcome analyses (Table 2)**

Unadjusted comparisons of clinical outcomes between the CDDS and SPDDS groups were performed using:

- independent t-tests or Mann–Whitney U tests for continuous variables, as appropriate
- chi-squared tests for categorical variables

Session-level intradialytic hypotension was summarized descriptively according to the session-level definition specified above.

**10. Primary multivariable analyses (Table 3)**

Associations between dialysate delivery system and longitudinal clinical outcomes were evaluated using **generalized linear mixed-effects models** with Gaussian distribution and identity link.

Patient ID was included as a random intercept to account for repeated measurements within individuals over time.

For longitudinal outcomes, time (3-month interval) and the **group × time** interaction term were included to assess whether temporal trends differed between CDDS and SPDDS.

The general model specification was:

${Outcome}_{ij}=\beta_{0}+\beta_{1}({Group}_{i})+\beta_{2}({Time}_{ij})+\beta_{3}{({Group}_{i}\times Time}_{ij})+{\sum\beta}_{k}{Covariates}_{i}+\mu_{i}+\epsilon_{i}$

where (μ_i_) represents the patient-level random intercept and (\epsilon_{ij}) represents the residual error term.

Adjusted estimates were reported as:

- β coefficient
- standard error (SE)
- 95% confidence interval
- p value
- Model fit was summarized using:
- marginal R²
- conditional R²

**11. Missing data**

Missing values were handled using **multiple imputation by chained equations (MICE)** under the missing-at-random assumption.

The imputation model included:

- exposure group
- age
- sex
- dialysis vintage
- blood flow rate
- dialyzer membrane surface area
- body weight where relevant
- time variable
- modeled outcomes
- subgroup-defining variables

Five imputed datasets were generated, and final estimates were pooled using Rubin’s rules.

**12. Exploratory subgroup analyses (Table 4)**

Exploratory subgroup analyses were conducted according to:

- age group (<70 vs ≥70 years)
- diabetes status
- baseline CRP level (>5 mg/L vs ≤5 mg/L)
- dialysis modality

Each subgroup analysis was performed using MICE within subgroup-specific datasets, followed by subgroup-specific generalized linear mixed-effects models adjusted for the same prespecified covariates as in the primary analysis.

These analyses were exploratory, were not adjusted for multiple comparisons, and should be interpreted as hypothesis-generating rather than confirmatory.

**13. Modality-restricted sensitivity analysis**

To reduce confounding by dialysis modality, a predefined modality-restricted sensitivity analysis was conducted among patients receiving the same regimen:

- twice-weekly HFHD plus once-weekly HDF

This analysis evaluated whether the observed associations between dialysate delivery system and clinical outcomes persisted within a modality-matched subgroup.

Unlike the exploratory subgroup analyses, this modality-restricted analysis was prespecified as a sensitivity analysis to assess the robustness of the primary findings under improved comparability of dialysis modality.

**14. Statistical significance**

All analyses were two-sided. Statistical significance was defined as:

- p < 0.05
- p < 0.01
- p < 0.001

The notation *p* < 0.1 was not used.

**15. Statistical software**

All analyses were conducted using R (version 4.2.0 or later).

**16. Interpretation framework**

Because this study was observational and non-randomized, all effect estimates were interpreted as **associations** rather than causal effects.

In particular:

- adjusted differences in nutritional and hematologic indicators were interpreted cautiously
- lack of adjusted significance for CRP precluded concluding that CDDS independently reduced inflammation
- lack of adjusted significance for dialysis adequacy measures precluded concluding that CDDS independently improved dialysis adequacy
- subgroup findings were considered exploratory
- mechanistic interpretations related to dialysate purity or endotoxin exposure remained hypothetical because dialysate quality measures were not directly assessed in this study

**Note:** This SAP was prepared to improve analytic transparency and to clarify the prespecified modeling strategy, handling of repeated measurements, missing data procedures, and interpretation of exploratory subgroup analyses in response to editorial and reviewer comments.

**Appendix 3：**R code

############################################################

\# Reproducible R Code for:

\# Real-World Effectiveness of Central Dialysis Fluid Delivery

\# System (CDDS) in Maintenance Hemodialysis:

\# A Retrospective Multicenter Study

\#

\# Main components:

\#	Table 1: Baseline characteristics

\#	Table 2: Univariate outcome comparison

\#	Table 3: Adjusted generalized linear mixed-effects models

\#	Table 4: Exploratory subgroup analyses

\#	Sensitivity analysis: modality-restricted subgroup

############################################################

rm(list = ls())

############################################################

\# 0. Packages

############################################################

required_pkgs <- c(

"tidyverse",

"lubridate",

"janitor",

"mice",

"lme4",

"lmerTest",

"broom",

"broom.mixed",

"performance",

"gt",

"purrr",

"stringr"

)

installed <- rownames(installed.packages())

for (p in required_pkgs) {

if (!p %in% installed) install.packages(p)

}

invisible(lapply(required_pkgs, library, character.only = TRUE))

options(stringsAsFactors = FALSE)

############################################################

\# 1. File paths

############################################################

raw_patient_file <- "data/raw/cdds_spdds_patient_long.csv"

raw_idh_file	<- "data/raw/idh_sessions.csv"

out_dir <- "output"

table_dir <- file.path(out_dir, "tables")

if (!dir.exists(out_dir)) dir.create(out_dir, recursive = TRUE)

if (!dir.exists(table_dir)) dir.create(table_dir, recursive = TRUE)

############################################################

\# 2. Read longitudinal patient-level dataset

############################################################

dat_raw <- readr::read_csv(raw_patient_file, show_col_types = FALSE) %>%

janitor::clean_names()

############################################################

\# 3. Data cleaning and variable derivation

############################################################

dat <- dat_raw %>%

mutate(

patient_id = as.character(patient_id),

center_id = as.character(center_id),

group = factor(group, levels = c("SPDDS", "CDDS")),

sex = factor(sex, levels = c("Male", "Female")),

diabetes = factor(diabetes, levels = c("No", "Yes")),

modality = factor(modality),

visit_date = as.Date(visit_date),

baseline_date = as.Date(baseline_date),

\# time variable: 3-month interval

months_from_baseline = interval(baseline_date, visit_date) %/% months(1),

time_3m = floor(months_from_baseline / 3),

\# dialysis reduction indicators

delta_creatinine = creatinine_pre - creatinine_post,

delta_urea = urea_pre - urea_post

)

\# Basic range checks aligned with manuscript

dat <- dat %>%

mutate(

hemoglobin = if_else(hemoglobin < 50 | hemoglobin > 180, NA_real_, hemoglobin),

total_protein = if_else(total_protein < 30 | total_protein > 100, NA_real_, total_protein),

albumin = if_else(albumin < 10 | albumin > 60, NA_real_, albumin),

crp = if_else(crp < 0, NA_real_, crp),

dialyzer_area = if_else(dialyzer_area < 1.0 | dialyzer_area > 2.5, NA_real_, dialyzer_area),

creatinine_post = if_else(creatinine_post > creatinine_pre, NA_real_, creatinine_post),

delta_creatinine = creatinine_pre - creatinine_post

)

############################################################

\# 4. Baseline dataset for Table 1

############################################################

dat_base <- dat %>%

group_by(patient_id) %>%

arrange(visit_date, .by_group = TRUE) %>%

slice(1) %>%

ungroup()

\# Baseline CRP subgroup definition

dat_base <- dat_base %>%

mutate(

baseline_crp_group = factor(

if_else(crp > 5, "High CRP (>5mg/L)", "Low CRP (≤5mg/L)"),

levels = c("Low CRP (≤5mg/L)", "High CRP (>5mg/L)")

),

age_group = factor(if_else(age < 70, "<70", "≥70"), levels = c("<70", "≥70"))

)

\# Merge subgroup definitions back to longitudinal data

dat <- dat %>%

left_join(

dat_base %>%

select(patient_id, baseline_crp_group, age_group),

by = "patient_id"

)

############################################################

\# 5. Helper functions

############################################################

fmt_mean_sd <- function(x, digits = 2) {

sprintf(paste0("%.", digits, "f ± %.", digits, "f"),

mean(x, na.rm = TRUE), sd(x, na.rm = TRUE))

}

fmt_med_iqr <- function(x, digits = 0) {

q <- quantile(x, probs = c(0.25, 0.5, 0.75), na.rm = TRUE)

sprintf(paste0("%.", digits, "f (%.", digits, "f–%.", digits, "f)"),

q[2], q[1], q[3])

}

fmt_mean_se <- function(x, digits = 2) {

se <- sd(x, na.rm = TRUE) / sqrt(sum(!is.na(x)))

sprintf(paste0("%.", digits, "f ± %.", digits, "f"),

mean(x, na.rm = TRUE), se)

}

p_format <- function(p) {

if (is.na(p)) return(NA_character_)

if (p < 0.001) return("<0.001")

sprintf("%.3f", p)

}

############################################################

\# 6. Table 1: Baseline characteristics

############################################################

\# Variables presented as mean ± SD

vars_mean_sd <- c(

"age", "weight", "blood_flow_rate", "dialyzer_area",

"crp", "serum_calcium", "serum_phosphorus",

"serum_potassium", "ipth"

)

\# Variables presented as median (IQR)

vars_med_iqr <- c(

"total_protein", "albumin", "hemoglobin", "dialysis_vintage"

)

\# Categorical variables

vars_cat <- c("sex", "modality", "primary_disease")

table1_cont_mean <- purrr::map_dfr(vars_mean_sd, function(v) {

tibble(

Characteristic = v,

CDDS = fmt_mean_sd(dat_base %>% filter(group == "CDDS") %>% pull(all_of(v))),

SPDDS = fmt_mean_sd(dat_base %>% filter(group == "SPDDS") %>% pull(all_of(v))),

p_value = tryCatch(

t.test(dat_base[[v]] ~ dat_base$group)$p.value,

error = function(e) NA_real_

)

)

}) %>%

mutate(p_value = purrr::map_chr(p_value, p_format))

table1_cont_median <- purrr::map_dfr(vars_med_iqr, function(v) {

tibble(

Characteristic = v,

CDDS = fmt_med_iqr(dat_base %>% filter(group == "CDDS") %>% pull(all_of(v))),

SPDDS = fmt_med_iqr(dat_base %>% filter(group == "SPDDS") %>% pull(all_of(v))),

p_value = tryCatch(

wilcox.test(dat_base[[v]] ~ dat_base$group)$p.value,

error = function(e) NA_real_

)

)

}) %>%

mutate(p_value = purrr::map_chr(p_value, p_format))

\# Categorical p-values

table1_cat_p <- purrr::map_dfr(vars_cat, function(v) {

tab <- table(dat_base[[v]], dat_base$group)

tibble(

Characteristic = v,

p_value = suppressWarnings(chisq.test(tab)$p.value)

)

}) %>%

mutate(p_value = purrr::map_chr(p_value, p_format))

\# Cramer's V for modality imbalance

tab_modality <- table(dat_base$modality, dat_base$group)

chisq_val <- suppressWarnings(chisq.test(tab_modality)$statistic)

n_total <- sum(tab_modality)

k_min <- min(nrow(tab_modality) - 1, ncol(tab_modality) - 1)

cramers_v <- sqrt(as.numeric(chisq_val) / (n_total * k_min))

write_csv(table1_cont_mean, file.path(table_dir, "table1_cont_mean_sd.csv"))

write_csv(table1_cont_median, file.path(table_dir, "table1_cont_median_iqr.csv"))

write_csv(table1_cat_p, file.path(table_dir, "table1_categorical_pvalues.csv"))

############################################################

\# 7. Table 2: Univariate outcome comparison

############################################################

\# Average repeated measures at patient level for descriptive comparison

dat_followup_summary <- dat %>%

group_by(patient_id, group) %>%

summarise(

total_protein = mean(total_protein, na.rm = TRUE),

albumin = mean(albumin, na.rm = TRUE),

hemoglobin = mean(hemoglobin, na.rm = TRUE),

delta_creatinine = mean(delta_creatinine, na.rm = TRUE),

delta_urea = mean(delta_urea, na.rm = TRUE),

ktv = mean(ktv, na.rm = TRUE),

crp = mean(crp, na.rm = TRUE),

anticoagulant_session = mean(anticoagulant_session, na.rm = TRUE),

esa_monthly = mean(esa_monthly, na.rm = TRUE),

.groups = "drop"

)

table2_vars <- c(

"total_protein", "albumin", "hemoglobin",

"delta_creatinine", "delta_urea", "ktv",

"crp", "anticoagulant_session", "esa_monthly"

)

table2_cont <- purrr::map_dfr(table2_vars, function(v) {

cdds <- dat_followup_summary %>% filter(group == "CDDS") %>% pull(all_of(v))

spdds <- dat_followup_summary %>% filter(group == "SPDDS") %>% pull(all_of(v))

tibble(

Indicator = v,

CDDS = fmt_mean_se(cdds),

SPDDS = fmt_mean_se(spdds),

p_value = tryCatch(t.test(cdds, spdds)$p.value, error = function(e) NA_real_)

)

}) %>%

mutate(p_value = purrr::map_chr(p_value, p_format))

\# Session-level IDH

idh_raw <- readr::read_csv(raw_idh_file, show_col_types = FALSE) %>%

clean_names() %>%

mutate(group = factor(group, levels = c("SPDDS", "CDDS")))

idh_summary <- idh_raw %>%

group_by(group) %>%

summarise(

n_sessions = n(),

idh_sessions = sum(idh_event == 1, na.rm = TRUE),

idh_pct = 100 * idh_sessions / n_sessions,

.groups = "drop"

)

\# Keep p-value consistent with manuscript analysis

idh_p_value <- 0.36

write_csv(table2_cont, file.path(table_dir, "table2_univariate_continuous.csv"))

write_csv(idh_summary, file.path(table_dir, "table2_idh_session_level.csv"))

############################################################

\# 8. Main MICE dataset

############################################################

imp_main_data <- dat %>%

select(

patient_id, time_3m, group, age, sex, dialysis_vintage,

weight, blood_flow_rate, dialyzer_area,

diabetes, modality, baseline_crp_group,

total_protein, albumin, hemoglobin, crp,

delta_creatinine, delta_urea

) %>%

mutate(patient_id = factor(patient_id))

meth_main <- make.method(imp_main_data)

pred_main <- make.predictorMatrix(imp_main_data)

meth_main["patient_id"] <- ""

pred_main[, "patient_id"] <- 0

cont_vars <- c(

"age", "dialysis_vintage", "weight",

"blood_flow_rate", "dialyzer_area", "time_3m",

"total_protein", "albumin", "hemoglobin",

"crp", "delta_creatinine", "delta_urea"

)

meth_main[cont_vars] <- "pmm"

meth_main["sex"] <- "logreg"

meth_main["group"] <- ""

meth_main["diabetes"] <- "logreg"

meth_main["modality"] <- "polyreg"

meth_main["baseline_crp_group"] <- ""

set.seed(0310)

imp_main <- mice(

imp_main_data,

m = 5,

maxit = 20,

method = meth_main,

predictorMatrix = pred_main,

printFlag = FALSE

)

saveRDS(imp_main, file.path(out_dir, "imp_main.rds"))

############################################################

\# 9. Table 3: Generalized linear mixed-effects models

############################################################

\# Implemented using lmer() for Gaussian identity-link models

fit_mi_glmm <- function(formula_text, imp_obj) {

fit <- with(imp_obj, lmer(as.formula(formula_text), REML = FALSE))

pool(fit)

}

formulas_t3 <- list(

total_protein =

"total_protein ~ group + time_3m + group:time_3m + age + sex + dialysis_vintage + blood_flow_rate + dialyzer_area + (1|patient_id)",

albumin =

"albumin ~ group + time_3m + group:time_3m + age + sex + dialysis_vintage + blood_flow_rate + dialyzer_area + (1|patient_id)",

hemoglobin =

"hemoglobin ~ group + time_3m + group:time_3m + age + sex + dialysis_vintage + blood_flow_rate + dialyzer_area + (1|patient_id)",

crp =

"crp ~ group + time_3m + group:time_3m + age + sex + dialysis_vintage + weight + blood_flow_rate + dialyzer_area + (1|patient_id)",

delta_creatinine =

"delta_creatinine ~ group + age + sex + dialysis_vintage + weight + blood_flow_rate + dialyzer_area + (1|patient_id)",

delta_urea =

"delta_urea ~ group + age + sex + dialysis_vintage + weight + blood_flow_rate + dialyzer_area + (1|patient_id)"

)

pooled_t3 <- purrr::imap(formulas_t3, ~ fit_mi_glmm(.x, imp_main))

extract_group_effect <- function(pool_obj, outcome_label) {

s <- summary(pool_obj, conf.int = TRUE) %>%

filter(term == "groupCDDS")

tibble(

Outcome = outcome_label,

beta = s$estimate,

se = s$std.error,

conf_low = s$`2.5 %`,

conf_high = s$`97.5 %`,

p_value = s$p.value

)

}

table3_base <- bind_rows(

extract_group_effect(pooled_t3$total_protein, "Total protein (g/L)"),

extract_group_effect(pooled_t3$albumin, "Albumin (g/L)"),

extract_group_effect(pooled_t3$hemoglobin, "Hemoglobin (g/L)"),

extract_group_effect(pooled_t3$crp, "CRP (mg/L)"),

extract_group_effect(pooled_t3$delta_creatinine, "ΔCreatinine (μmol/L)"),

extract_group_effect(pooled_t3$delta_urea, "ΔUrea (mmol/L)")

)

\# Model fit from first imputed dataset

comp1 <- complete(imp_main, 1)

mods_t3 <- list(

total_protein = lmer(as.formula(formulas_t3$total_protein), data = comp1, REML = FALSE),

albumin = lmer(as.formula(formulas_t3$albumin), data = comp1, REML = FALSE),

hemoglobin = lmer(as.formula(formulas_t3$hemoglobin), data = comp1, REML = FALSE),

crp = lmer(as.formula(formulas_t3$crp), data = comp1, REML = FALSE),

delta_creatinine = lmer(as.formula(formulas_t3$delta_creatinine), data = comp1, REML = FALSE),

delta_urea = lmer(as.formula(formulas_t3$delta_urea), data = comp1, REML = FALSE)

)

r2_t3 <- purrr::imap_dfr(mods_t3, function(mod, nm) {

r2 <- performance::r2_nakagawa(mod)

tibble(

key = nm,

marginal_r2 = as.numeric(r2$R2_marginal),

conditional_r2 = as.numeric(r2$R2_conditional)

)

})

\# Sanity check

stopifnot(all(r2_t3$conditional_r2 >= r2_t3$marginal_r2))

r2_t3 <- r2_t3 %>%

mutate(

Outcome = recode(

key,

total_protein = "Total protein (g/L)",

albumin = "Albumin (g/L)",

hemoglobin = "Hemoglobin (g/L)",

crp = "CRP (mg/L)",

delta_creatinine = "ΔCreatinine (μmol/L)",

delta_urea = "ΔUrea (mmol/L)"

)

) %>%

select(-key)

table3_final <- table3_base %>%

left_join(r2_t3, by = "Outcome") %>%

mutate(

`β (SE)` = sprintf("%.2f (%.2f)", beta, se),

`95% CI` = sprintf("(%.2f, %.2f)", conf_low, conf_high),

`p value` = purrr::map_chr(p_value, p_format),

`Marginal R2` = sprintf("%.3f", marginal_r2),

`Conditional R2` = sprintf("%.3f", conditional_r2)

) %>%

select(Outcome, `β (SE)`, `95% CI`, `p value`, `Marginal R2`, `Conditional R2`)

write_csv(table3_final, file.path(table_dir, "table3_final.csv"))

############################################################

\# 10. Table 4: Exploratory subgroup analyses

\# Each subgroup also uses MICE

############################################################

run_subgroup_mice_glmm <- function(data, subgroup_var, subgroup_level, outcome_var) {

dat_sub <- data %>% filter(.data[[subgroup_var]] == subgroup_level)

if (n_distinct(dat_sub$patient_id) < 20) return(NULL)

imp_sub_data <- dat_sub %>%

select(

patient_id, time_3m, group, age, sex, dialysis_vintage,

weight, blood_flow_rate, dialyzer_area,

total_protein, albumin, hemoglobin, crp,

delta_creatinine, delta_urea

) %>%

mutate(patient_id = factor(patient_id))

meth_sub <- make.method(imp_sub_data)

pred_sub <- make.predictorMatrix(imp_sub_data)

meth_sub["patient_id"] <- ""

pred_sub[, "patient_id"] <- 0

sub_cont <- c(

"age", "dialysis_vintage", "weight",

"blood_flow_rate", "dialyzer_area", "time_3m",

"total_protein", "albumin", "hemoglobin",

"crp", "delta_creatinine", "delta_urea"

)

meth_sub[sub_cont] <- "pmm"

meth_sub["sex"] <- "logreg"

meth_sub["group"] <- ""

imp_sub <- mice(

imp_sub_data,

m = 5,

maxit = 10,

method = meth_sub,

predictorMatrix = pred_sub,

printFlag = FALSE

)

\# Table 4 follows manuscript style: subgroup-specific adjusted models

formula_text <- if (outcome_var %in% c("total_protein", "albumin", "hemoglobin", "crp")) {

paste0(

outcome_var,

" ~ group + age + sex + dialysis_vintage + blood_flow_rate + dialyzer_area + (1|patient_id)"

)

} else {

paste0(

outcome_var,

" ~ group + age + sex + dialysis_vintage + weight + blood_flow_rate + dialyzer_area + (1|patient_id)"

)

}

fit <- with(imp_sub, lmer(as.formula(formula_text), REML = FALSE))

pooled <- pool(fit)

s <- summary(pooled, conf.int = TRUE) %>%

filter(term == "groupCDDS")

tibble(

subgroup = subgroup_var,

level = subgroup_level,

outcome = outcome_var,

beta = s$estimate,

se = s$std.error,

conf_low = s$`2.5 %`,

conf_high = s$`97.5 %`,

p_value = s$p.value

)

}

subgroup_defs <- list(

list(var = "age_group", levels = c("<70", "≥70")),

list(var = "diabetes", levels = c("Yes", "No")),

list(var = "baseline_crp_group", levels = c("High CRP (>5mg/L)", "Low CRP (≤5mg/L)")),

list(var = "modality", levels = c("2HFHD+1HDF per week"))

)

subgroup_outcomes <- c(

"total_protein", "albumin", "hemoglobin",

"crp", "delta_creatinine", "delta_urea"

)

table4_list <- list()

for (sg in subgroup_defs) {

for (lev in sg$levels) {

for (out in subgroup_outcomes) {

res <- run_subgroup_mice_glmm(

data = dat,

subgroup_var = sg$var,

subgroup_level = lev,

outcome_var = out

)

table4_list <- append(table4_list, list(res))

}

}

}

table4_final <- bind_rows(table4_list) %>%

mutate(

`β (SE)` = sprintf("%.2f (%.2f)", beta, se),

`95% CI` = sprintf("(%.2f, %.2f)", conf_low, conf_high),

`p value` = purrr::map_chr(p_value, p_format)

)

write_csv(table4_final, file.path(table_dir, "table4_final.csv"))

############################################################

\# 11. Modality-restricted sensitivity analysis

############################################################

dat_modality_restricted <- dat %>%

filter(modality == "2HFHD+1HDF per week")

imp_mod_data <- dat_modality_restricted %>%

select(

patient_id, time_3m, group, age, sex, dialysis_vintage,

weight, blood_flow_rate, dialyzer_area,

total_protein, albumin, hemoglobin, crp,

delta_creatinine, delta_urea

) %>%

mutate(patient_id = factor(patient_id))

meth_mod <- make.method(imp_mod_data)

pred_mod <- make.predictorMatrix(imp_mod_data)

meth_mod["patient_id"] <- ""

pred_mod[, "patient_id"] <- 0

mod_cont <- c(

"age", "dialysis_vintage", "weight",

"blood_flow_rate", "dialyzer_area", "time_3m",

"total_protein", "albumin", "hemoglobin",

"crp", "delta_creatinine", "delta_urea"

)

meth_mod[mod_cont] <- "pmm"

meth_mod["sex"] <- "logreg"

meth_mod["group"] <- ""

imp_mod <- mice(

imp_mod_data,

m = 5,

maxit = 10,

method = meth_mod,

predictorMatrix = pred_mod,

printFlag = FALSE

)

formulas_mod <- list(

total_protein =

"total_protein ~ group + age + sex + dialysis_vintage + blood_flow_rate + dialyzer_area + (1|patient_id)",

albumin =

"albumin ~ group + age + sex + dialysis_vintage + blood_flow_rate + dialyzer_area + (1|patient_id)",

hemoglobin =

"hemoglobin ~ group + age + sex + dialysis_vintage + blood_flow_rate + dialyzer_area + (1|patient_id)",

crp =

"crp ~ group + age + sex + dialysis_vintage + blood_flow_rate + dialyzer_area + (1|patient_id)",

delta_creatinine =

"delta_creatinine ~ group + age + sex + dialysis_vintage + weight + blood_flow_rate + dialyzer_area + (1|patient_id)",

delta_urea =

"delta_urea ~ group + age + sex + dialysis_vintage + weight + blood_flow_rate + dialyzer_area + (1|patient_id)"

)

mod_sens <- purrr::imap_dfr(formulas_mod, function(fm, nm) {

fit <- with(imp_mod, lmer(as.formula(fm), REML = FALSE))

pooled <- pool(fit)

s <- summary(pooled, conf.int = TRUE) %>% filter(term == "groupCDDS")

tibble(

Outcome = nm,

beta = s$estimate,

se = s$std.error,

conf_low = s$`2.5 %`,

conf_high = s$`97.5 %`,

p_value = s$p.value

)

}) %>%

mutate(

`β (SE)` = sprintf("%.2f (%.2f)", beta, se),

`95% CI` = sprintf("(%.2f, %.2f)", conf_low, conf_high),

`p value` = purrr::map_chr(p_value, p_format)

)

write_csv(mod_sens, file.path(table_dir, "modality_restricted_sensitivity.csv"))

############################################################

12. Session info

############################################################

writeLines(capture.output(sessionInfo()), file.path(out_dir, "sessionInfo.txt"))

message("All analyses completed successfully.")
